# ACE2 Serum Levels as Predictor of Infectability and Outcome in COVID-19

**DOI:** 10.3389/fimmu.2022.836516

**Published:** 2022-03-23

**Authors:** María del Carmen Maza, María Úbeda, Pilar Delgado, Lydia Horndler, Miguel A. Llamas, Hisse M. van Santen, Balbino Alarcón, David Abia, Laura García-Bermejo, Sergio Serrano-Villar, Ugo Bastolla, Manuel Fresno

**Affiliations:** ^1^ Centro de Biología Molecular Severo Ochoa, Consejo Superior de Investigaciones Científicas (CSIC), Universidad Autónoma de Madrid, Madrid, Spain; ^2^ Instituto Sanitario Princesa, Madrid, Spain; ^3^ EMPIREO Diagnóstico Molecular Sociedad Limitada (SL), Madrid, Spain; ^4^ Hospital Universitario Ramón y Cajal, Instituto Ramón y Cajal de Investigación Sanitaria (IRYCIS), Universidad de Alcalá, Madrid, Spain

**Keywords:** COVID-19, ACE2, antibodies, neutralization, biomarker

## Abstract

**Background:**

COVID‐19 can generate a broad spectrum of severity and symptoms. Many studies analysed the determinants of severity but not among some types of symptoms. More importantly, very few studies analysed patients highly exposed to the virus that nonetheless remain uninfected.

**Methods:**

We analysed serum levels of ACE2, Angiotensin II and anti-Spike antibodies in 2 different cohorts at high risk of viral exposure, highly exposed but uninfected subjects, either high risk health care workers or persons cohabiting with infected close relatives and seropositive patients with symptoms. We tested the ability of the sera of these subjects to neutralize lentivirus pseudotyped with the Spike-protein.

**Results:**

We found that the serum levels of ACE2 are significantly higher in highly exposed but uninfected subjects. Moreover, sera from this seronegative persons can neutralize SARS-CoV-2 infection in cellular assays more strongly that sera from non-exposed negative controls eventhough they do not have anti-CoV-2 IgG antibodies suggesting that high levels of ACE2 in serum may somewhat protect against an active infection without generating a conventional antibody response. Finally, we show that among patients with symptoms, ACE2 levels were significantly higher in infected patients who developed cutaneous as compared with respiratory symptoms and ACE2 was also higher in those with milder symptoms.

**Conclusions:**

These findings suggest that soluble ACE2 could be used as a potential biomarker to predict SARS-CoV-2 infection risk and to discriminate COVID-19 disease subtypes.

## Introduction

The causative agent of Coronavirus disease 2019 (COVID-19), Severe Acute Respiratory Syndrome Coronavirus-2 (SARS-CoV-2), enter to human cells *via* the receptor-binding domain (RBD) of its spike (S) protein that interacts with the angiotensin-converting enzyme 2 (ACE2) receptor (1, 2). ACE2 is a membrane bound enzyme expressed in numerous different cell types and tissues such as lungs, arteries, heart, and intestine. ACE2 catalyzes the cleavage of angiotensin II (AngII) into angiotensin 1-7, regulating the renin-angiotensin-aldosterone system (RAS), playing an important physiologic role in the homeostasis of tissue microcirculation and inflammation (3, 4).

COVID‐19 can generate a broad spectrum of severity and symptoms, whose reason is not completely understood yet. The severity of COVID-19 ranges from asymptomatic cases to fatal pneumonia (5). Around 90% of infected people experience weak or no symptoms while other patients require ICU care and may die. The death rate increases dramatically with age and is higher in men than in women.

Besides, different symptoms in COVID-19 have been described with involvement not only in the lung, but also in the gastrointestinal tract or vascular tissues (6, 7). In addition, cutaneous manifestations in patients with concurrent dermatitis and COVID-19 infection has been described form early times in the pandemic (8).

Therefore, good diagnostic/prognostic tools are required to guide clinical decision-making in COVID-19 by allowing to predict severity and symptoms of the disease. Many Biomarkers for predicting prognosis of disease have been described see e.g (9). Severe or critical COVID-19 is characterised by increased innate immune response, decreased adaptive immune response, as well as increased markers of tissue damage and major organ failure (10) as well as metabolic markers (11, 12). Most of them are used for managing severe ill patients or to understand why some patients develop mild symptoms but few of them have discriminated the types of symptoms.

Moreover, despite the high transmissibility of SARS-CoV-2, there are highly exposed people who have not acquired the infection. Genetic factors along with other risk factors can determine the susceptibility of each individual to infection, but those are largely unknown (13, 14). The goal of our study is to provide some insight into why health care worker (HCW) with reported high-risk exposures to virus and persons cohabiting without protection with infected relatives remained uninfected despite constant contact with the virus. We have found significantly higher soluble serum ACE2 levels in highly exposed but uninfected seronegative subjects, either HCW, daily dealing with COVID-19 patients in the hospital or persons cohabiting with infected close relatives, compared to infected seropositive patients. Furthermore, we demonstrated that serum from highly exposed but uninfected subjects have the capacity of neutralize SARS-CoV-2, probably mediated by soluble ACE2. Finally, focus on infected patients also revealed differences in ACE2 levels and anti-S IgG1 titters in the diverse groups of infected patients. In particular, higher ACE2 levels are present in patients who developed cutaneous symptoms in contrast to those with pneumonia or other respiratory involvement and in general, we observed lower, although only marginally significant, ACE2 levels in more severe patients.

## Material and Methods

### Patients and Sample Collection

All participants provided written consent to participate in the study, which was performed according to the EU guidelines and following the ethical principles of the Declaration of Helsinki. A total of 147 human sera were obtained from volunteers that contacted EMPIREO SL (www.empireo.es) for serological tests and were included after written informed consent. The following commercial serological tests were assayed: Feal Test of Hangzhou Alltest Biotech, reference number RPP25COV1925; Sienna Test of Salofa Oy, reference number 102221; BiosSynex Test of Biosynex Swiss, reference number SW40005; Vircell ELISA test of Vircell SL, reference number G1032.

Serum donors filled in a questionnaire to allow their clinical classification according to the following parameters: Asymptomatic, no symptoms; Mild, 3 or less of the following symptoms: non-productive cough, hyperthermia, headache, odynophagia, dyspnea, asthenia, myalgia, ageusia, anosmia, cutaneous involvement; Moderate, 3 or more of the above symptoms plus gastrointestinal symptoms, or more than 3 of the above for 7 or more days; Severe, pneumonia requiring hospitalization and intubation (15). The study protocol was approved by Empireo internal Committees as well as by Universidad Autonoma de Madrid (CEI-117- 2352).

A second set of 30 serum samples was obtained from HCW, who had been on duty for at least three months in COVID19 wards or intensive care units of the Ramón y Cajal Hospital in Madrid. They reported at least three high-risk exposures to SARS-CoV-2 (16) without having experienced symptoms suggestive of SARS-CoV-2 infection. The absence of infection was confirmed by a negative PCR SARS-CoV-2 test and the absence of SARS-CoV-2 IgM and IgG in plasma by indirect chemiluminescence immunoassay (Vircell, Granada, Spain) -COVID-19 VIRCLIA IgM+IgA monotest, Vircell SL, reference number VCM09. The most frequent type of exposures were largely unprotected exposure to aerosol-generating procedures or patient secretions, and close contact without face masks with other confirmed cases of COVID-19. The study was carried out at the Ramón y Cajal University Hospital in Madrid (Spain) and was approved by the local Research Ethics Committee (ceic.hrc@salud.madrid. org, approval number 095/20).

### Anti-S Flow Cytometry Assay

Detection of conformational anti S antibodies was performed as described (15). Briefly, Jurkat cells stably expressing the Wuhan S variant (Jurkat-s) were incubated for 30 min on ice with 1:50 dilutions of human sera in phosphate-buffered saline (PBS), 1% bovine serum albumin (BSA), 0.02% sodium azide. Cells were centrifuged 2 times for 5 min at 500 g. The cell pellet was finally resuspended in a 1:300 dilution of mouse anti-human IgG1 Fc-PE (Ref.: 9054-09, Southern Biotech) and a 1:500 dilution of the Brilliant Violet 421™ anti-human EGFR Antibody (Ref.: 352911, Biolegend) in PBS-BSA-sodium azide. Samples were then washed and labeled cells were analyzed on a FACSCanto II flow cytometer (Becton-Dickinson) and the data were analyzed with FlowJo software (BD).

### ELISA

ACE2, AngII and ADAM17 were measured according to respective the manufacturer’s protocol kits (ab235649 Human ACE2 simple step ELISA kit, Abcam; Human Angiotensin II ELISA kit, Reddot Biotech; Human TACE/ADAM17 DuoSet ELISA kit, R&D Systems). The OD at 450nm was determined on a FLUOstar OPTIMA reader (BMG Labtech).

### Neutralization Assays

Lentiviral supernatants were produced from transfected HEK-293T cells as described previously (15). Briefly, lentiviruses were obtained by co-transfecting plasmids pCMV (gag/pol), pHRSIN-GFP and a truncated S envelope (pCR3.1-St) using the JetPEI transfection reagent (Polyplus Transfection). Viral supernatants were obtained after 48 hours post-transfection. Polybrene (8 µg/mL) was added to the viral supernatants prior to transduction of ACE2+HEK293T cells. A total of 35-50 x 10^3^ ACE2+HEK293T cells per p48 well were seeded the day before transduction. Serially diluted plasma or recombinant human ACE-2 Fc chimera (rhACE2, R&D Systems) were incubated with viral supernatant for 1 hour at 37 degrees prior to addition to the cells. Cells were left in culture for 48 hours, then were resuspended in PBS with 2% FBS and 5mM EDTA and fixed with 2% paraformaldehide. GFP+ cells were then analyzed on a FACSCalibur flow cytometer (Becton-Dickinson) and the data were analyzed with FlowJo software (BD).

In a second assay performed in biosafety level 3 (BSL3), a total of 20 x 10^4^ Calu-3 cells (2B4) per p24 well were seeded. The next day, serially diluted rhACE2 were incubated with authentic SARS-CoV-2 (MAD6) at 5x10^3^ cfu/ml for 1 hour at 37 degrees prior to addition to the cells. Cells were left with preincubated virus for 1hour, washed and cultured for 48hours. After the trizol RNA extraction, the presence of SARS-CoV-2 RNA in cells were determined by PCR using the N2 sequence primers (2019-nCoV_N2-F: TTACAAACATTGGCCGCAAA; 2019-nCoV_N2-R: GCGCGACATTCCGAAGAA) Data were normalized by 18S sequence primer (18S F: 5`GCAATTATTCCCCATGAACG; 18S R: 5`GGGACTTAATCAACGCAAGC).

### Statistical Analysis

Correlation coefficients were assessed with the t-test. Significant differences between two groups in ACE2 levels and other quantitative measures were assessed through the non parametric Mann-Whitney-Wilcoxon test (two tails). Groups were also compared by measuring the fraction of sample that fall above a high threshold (below a low threshold) and assessing the significant differences through binomial tests. In this way, we can consider non-symmatrical situations in which two samples are similar below the threshold but highly dissimilar above it, or viceversa.

## Results

### High Levels of Serum ACE2 Correlate With Lower Susceptibility to Infection

We analysed 2 different cohorts (for a total of 175 persons) that were at high risk of viral exposure (**Supplementary Table 1**). The first cohort consisted of persons cohabiting in a close familiar unit with one or more infected relatives for more than 2 months during the lock down in Spain from March to May 2020, without taking preventive measures at home. They were monitored at least twice at different times and with at 2-4 different serological tests to be defined as seronegative o seropositive, before analysis and sample collection. The second cohort consisted of serum samples collected from healthcare workers (HCW) (Hospital Ramón y Cajal, Madrid) who had been on duty for at least three months in COVID19 wards or intensive care units and reported at least three high-risk exposures to SARS-CoV-2 without having experienced symptoms suggestive of SARS-CoV-2 infection, persistently negative PCR SARS-CoV-2 testing, and absence of SARS-CoV-2 IgM and IgG in plasma.

Both groups were compared with corresponding seropositive infected controls. In all those persons who had been infected the serum samples were collected at least 50 days after the disappearance of symptoms to allow for full recovery of the homeostatic levels of ACE2/Ang II in each one of them. We also included samples from healthy volunteers unexposed to the virus and determined to be seronegative by more than one test, as well as a few non-COVID-19 patients but having similar respiratory symptoms to those associated with COVID-19. None of the subjects shows signs of any other pathology in the 45-60 days previous to blood collection.

**Figure 1:**
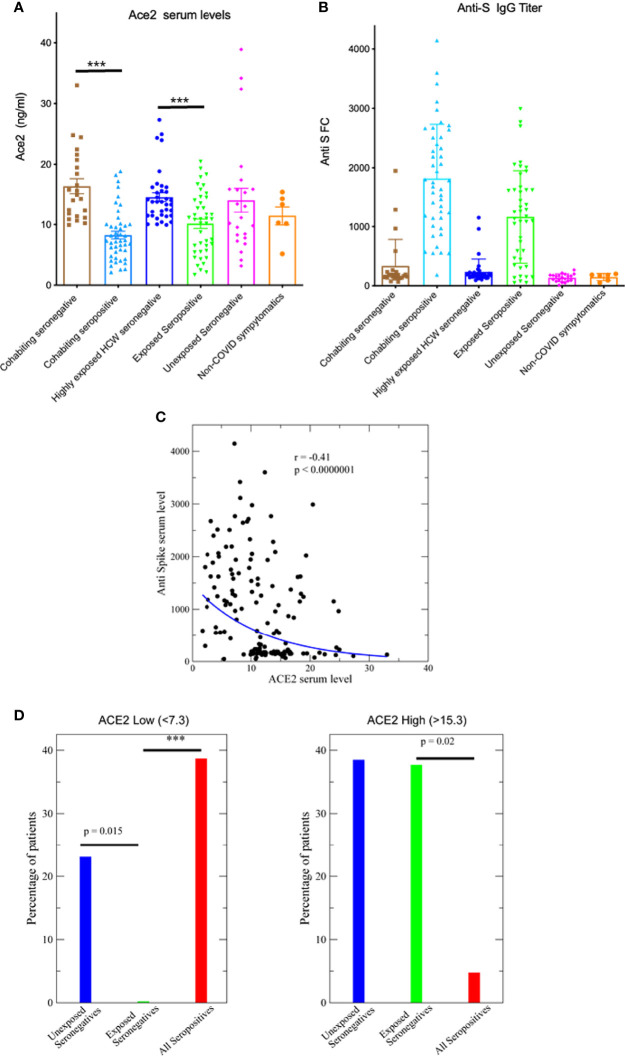
Serum ACE2 levels and susceptibility to infection. **(A)** ACE2 serum levels determined by ELISA in the indicated patient groups, ***p<0.0001. **(B)** Detection of anti-S protein IgG1 antibodies in human patient sera by flow cytometry of Jurkat cells stably expressing the full-length native S protein of SARS-CoV-2. **(C)** Anti-S IgG1 antibody is negatively related with ACE2 serum level, p<0.0000001. **(D)** The percentage of patients with low (ACE2<7.3, lowest 25% of all samples) and high levels of ACE2 (ACE2>12.3, highest 25% of all samples), ***p<0.0001.

Interestingly, significant differences were detected in the serum levels of ACE2. Disease-free unexposed seronegative controls have a wide range of serum ACE2 levels, ranging from 3 to 40 ng/ml. Interestingly, seronegative subjects highly exposed to the virus, either because of their close and daily living with infected relatives or because of their health work with COVID-19 patients, have significantly higher ACE2 levels than those who are seropositive with similar exposure conditions (p< 0.0001 in both cases, two-tailed Mann-Whitney test, as for the other indicated p-values unless otherwise stated). Interestingly, the levels of ACE2 in those exposed but seronegative cases were always higher than 10 ng/ml (**Figure 1A**). Non-COVID-19 patients with some symptoms similar to COVID patients had a similar distribution. We only observed small but not significant differences concerning age and sex of patients (44 vs. 47 years, p=0.37 and 42% vs. 47% females p=0.69 for seronegatives and seropositives, respectively). To further corroborate the serologic status of the subjects, we used a highly sensitive S-specific IgG1 antibody detection assay developed by us that uses a Jurkat cell line expressing the native form of the S protein (16). A few of the seronegatives by the clinical tests were tested as positive in this assay indicating the superior ability to detect infected people (**Figure 1B**). Besides the dichotomy between seropositive and seronegative samples, there is a significant negative correlation between ACE2 levels and the quantity of developed anti spike antibodies (r=-0.41, t=-5.3, **Figure 1C**).

To further corroborate the significance of ACE2 levels we analysed the fraction of seronegative and seropositive positive samples using a 25% threshold. Thresholds were chosen such that 25% of all ACE2 measures fall above (below) the threshold, obtaining 15.3 ng/ml as high threshold and 7.3 ng/ml as low threshold. The fraction with ACE2<7.3 ng/ml is zero for exposed negative samples, while it is 39% for samples from seropositive patients and it is 23% for unexposed seronegative samples We tested whether the difference is significant through an exact binomial test, finding that the difference between exposed seronegative samples and unexposed seronegatives is indeed significant (p=0.015) (**Figure 1D**). The fraction in all exposed seronegative samples having low ACE2 is significantly lower than for unexposed seronegative (p<0.0001) (**Figure 1D**) whereas the fraction of exposed seronegative samples with high ACE2 is higher than for positive samples (p=0.02) (**Figure 1D**). Similar results are obtained with a different threshold for low and high ACE2 levels (20%) (not shown). Similarly, levels of Ang II were measured although no clear correlation was detected (**Supplementary Figure S1**). Therefore, these results suggest that high levels of ACE2 correlate with a lower risk of becoming infected.

### Serum of Highly Exposed Seronegative Subjects Has the Capacity to Neutralize SARS-CoV-2

**Figure 2:**
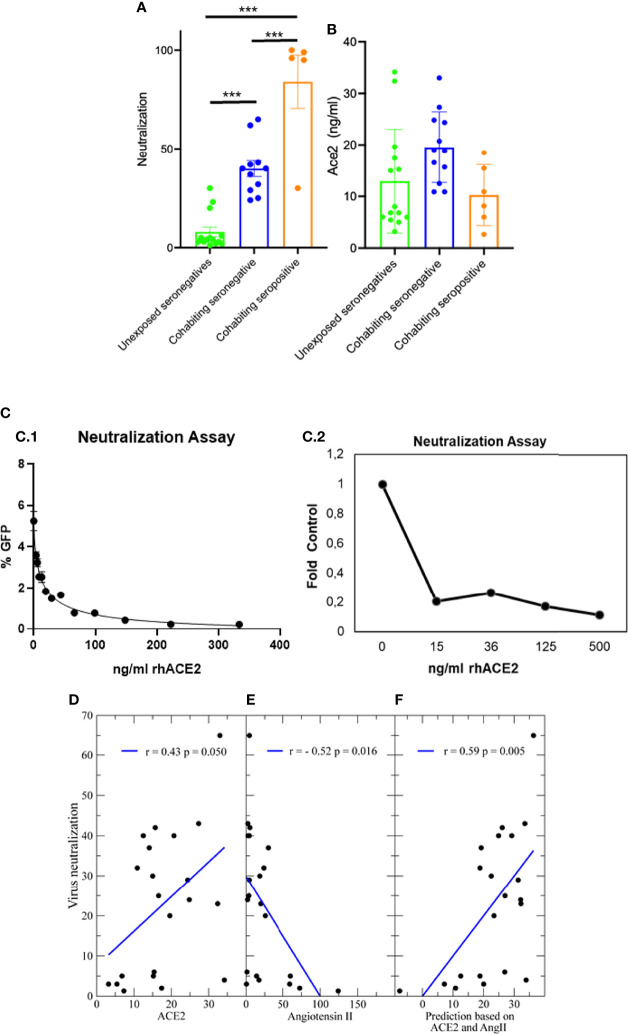
Neutralization of SARS-CoV-2. **(A)** Neutralization of a recombinant virus pseudotyped with the Spike protein and at ¼ dilution of all sera. Values are the mean of 2 different experiments. Neutralization versus subjects group, ***p<0.001 among the 3 groups. **(B)** ACE2 serum levels for the same samples. **(C)** Neutralization by soluble hACE2 of a recombinant virus pseudotyped with the Spike protein (C1) and authentic SARS-CoV2 (C2). **(D)** There is a significant positive correlation between neutralization and ACE2 **(E)** and negative correlation between neutralization and Angiotensin II, which binds ACE2 and might compete for the binding of viral particles. **(F)** A regularized linear fit that takes into account both ACE2 and Angiotensin II explains little more than one third of the variance of the neutralization (p=0.005).

In order to explain why subjects in contact with the virus remained uninfected, we investigated the ability of lentivirus pseudotyped with the Spike-protein to infect ACE2 expressing cells in the presence of the sera of these subjects. Interestingly, sera obtained from highly exposed seronegative patients either HCW or cohabiting with infected relatives despite having no antibodies against S protein, neutralized significantly better (p<0.001) than sera from negative controls. Highly exposed seronegative sera have a detectable neutralizing activity at ¼ dilution (**Figure 2A**), significantly higher (p<0.001) than that of sera from unexposed seronegative control subjects that was much lower and in many cases undetectable. As expected, most of the seropositive patients tested, all of them with high titers of anti-S IgG1 antibodies, showed greater neutralization ability. This suggests that the serum of highly exposed people have an anti-S antibody-independent factor in the serum that may neutralize the virus. The ACE2 serum levels were on average higher in the neutralizing exposed seronegative sera that in unexposed seronegatives (**Figure 2B**).

Since ACE2 is the receptor for the virus, a possible explanation is that high soluble ACE2 titter in the serum of seronegative subjects would neutralize the virus by binding to protein S and inhibiting its entry into cells. In support of this interpretation, we have found that soluble recombinant human ACE2 prevents the entry into cells of virus pseudotyped to express the S protein in the same neutralization assay (**Figure 2C**1). A dose response curve with IC_50 =_ 11.90 ng/ml sACE2 was obtained. Interestingly, this value was close to the cut-off observed on sera levels for highly exposed seronegative patients (HCW or Cohabitants seronegative) since unexposed seronegative control samples with levels of sACE2 lower than 10ng/ml have almost negligible capacity to neutralize the virus. Interestingly, recombinant human ACE2 also block with similar sensitivity the infection of a pulmonary cell line, Calu-3, with infectious SARS-CoV2 (**Figure 2C**2).

In support of this hypothesis, the ability of sera to neutralize the virus is quantitatively related, although weakly, with the ACE2 level (**Figure 2D**, correlation r=0.43, t=2.1, n=21, p=0.05 only control and seronegative samples) and it is inversely related with the AngII level (**Figure 2E**, r=-0.52, t=-2.6, p<0.02), possibly because AngII competes with the spike protein of SARS-COV-2 for binding to ACE2. A linear model that takes into account both ACE2 and AngII, and that is strongly regularized through rescaled ridge regression to limit overfitting, explains more than one third of the variance of the neutralization (**Figure 2F**, r=0.59, t=3.1, p=0.05). Therefore, these results suggest that high levels of ACE2 in serum may somewhat protect against an active infection without generating a conventional antibody response.

### ACE2 Serum Levels Are Associated With the Type of Symptoms

**Figure 3:**
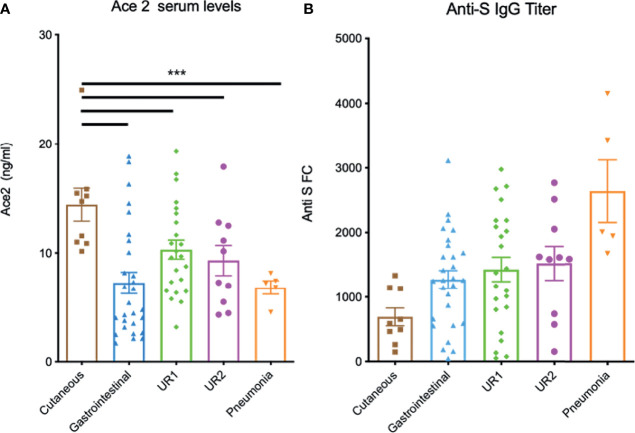
Serum ACE2 and anti-S IgG levels in infected seropositive patients according with the type of symptoms. **(A)** ACE2 serum levels determined by ELISA in the indicated patient groups ***p<0.001. **(B)** Detection of anti-S protein antibodies in human patient sera by flow cytometry of Jurkat cells stably expressing the full-length native S protein of SARS-CoV-2.

Next, seropositive serum donors were clinically classified according to the type (cutaneous, gastrointestinal or respiratory) and severity of symptoms as described in methods [see also (15)]. Indeed, those with respiratory symptoms were subdivided in 3 groups with different severity, mild (UR1) moderate (UR2) and pneumonia. Interestingly, when patients were classified according the type of symptoms, we observed a significant correlation between levels of ACE2 in serum and some symptoms. Infected patients developing cutaneous manifestations had significantly higher ACE2 levels than the rest (p<0.001) (**Figure 3A**) whereas patients with gastrointestinal symptoms tend to have lower ACE2 levels, although with a large dispersion. Patients with upper respiratory tract symptoms tend to have intermediate ACE2 levels, while patients with pneumonia tend to have lower ACE2 (**Figure 3A**). In contrast, conformational anti-S IgG1s were clearly the lowest in patients developing cutaneous manifestations and the highest in those with pneumonia (**Figure 3B**) consistent with the overall negative correlation between ACE2 and anti-S levels. We also analyzed serum levels of ADAM17 that has been suggested to be involved in the increase in plasma levels of sACE2 (17). Nevertheless, there was not significant differences between the groups (**Supplementary Figure S2**).

**Figure 4:**
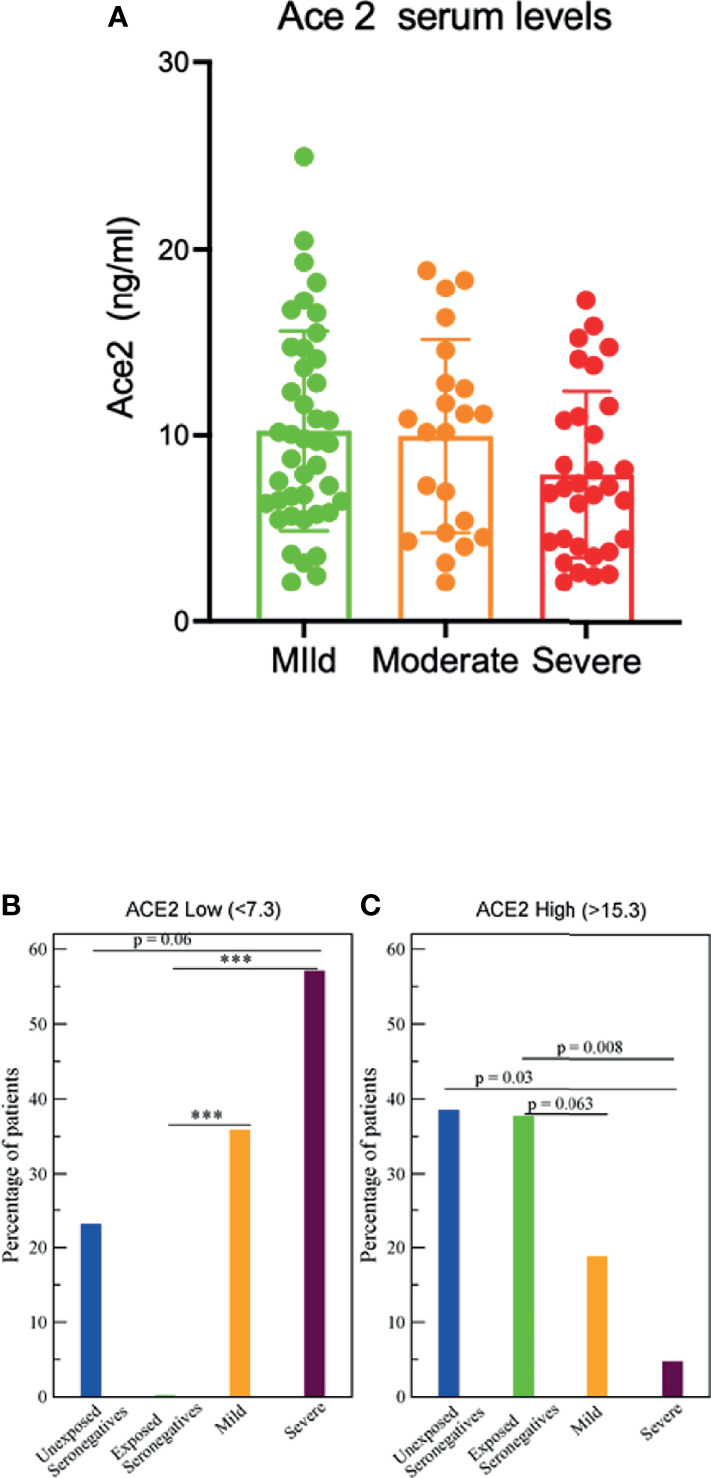
Serum ACE2 levels and severity. **(A)** ACE2 serum levels determined by ELISA in the indicated patients groups **(B)** The fraction of samples with low ACE2 (ACE2<7.3, lowest 25% of all samples), ***p<0.0001. **(C)** The fraction of samples with high ACE2 (ACE2>12.3, highest 25% of all samples).

Next, infected patients were classified according to the severity of symptoms regardless of their type (as described in methods and (15). In patients with milder symptoms ACE2 serum levels tended to be higher than in more severe patients (**Figure 4A**). To test whether there were some significant differences, we analyzed the 25% thresholds of low and high ACE2 levels 7.3 and 15.3, respectively, as mentioned above. Interestingly samples from severe patients with low ACE2 are much more frequent than for the control population of unexposed seronegatives (57% versus 23%, p=0.062) and especially than from exposed seronegatives (p<0.0001) (**Figure 4B**). Even infected patients with mild symptoms had a significant larger percent of samples than exposed seronegatives with levels higher than 7.3 (p<0.0001) (**Figure 4B**). We repeated these tests with a more stringent threshold of 20% (ACE2<6.8) with almost the same qualitative results.

The same analysis for the fraction of samples with ACE2 higher than 15.3 (25% level) is shown in **Figure 4C**. The lowest fraction is for samples from severe patients (p=0.008) respect to exposed seronegative samples whereas mild samples have also lower frequencies (p=0.03). The Mann-Whitney non parametric test shows that the ACE2 level of severe patients is marginally significantly lower than for mild patients (p=0.054). Altogether, the results supports that there is a negative relationship between low ACE2 levels and the severity of the symptoms.

## Discussion

Many studies have searched for biomarkers that can predict the prognosis of COVID-19, but very few studies addressed the susceptibility to infection of highly exposed individuals as HCW (18–20) and none, to our knowledge, of those persons cohabiting in close contacts with infected patients without taking any preventive measure. Here, we have addressed this question. We found that subjects with lower ACE2 would be infected with high probability than those with higher ACE2. Thus, higher levels of ACE2 in serum are associated with a certain innate resistance to undergo infection, at least a productive infection that gives rise to virus-specific IgG antibodies.

Besides this qualitative difference between highly exposed seronegative subjects and seropositive ones, there is a quantitative negative correlation between anti-S and ACE2 serum levels, suggesting that persons with high ACE2 serum levels need to develop lower antibody production to get rid of the infection, sometimes so low that it may be undetectable. This may therefore identify those who can better resist to be infected. Consistent with this view, patients that developed mild infection present higher ACE2 serum levels than those that developed severe infection. Our analysis strongly suggests that low serum ACE2 levels is a negative prognostic factor in the susceptibility to infection.

Moreover, we have been able to observe that ACE2 serum levels in those persons infected correlate with the type of symptoms and in lesser extent with the severity of the infection. Again, our study fills a gap, since most of the studies on biomarkers focused on severity rather than on the type of symptoms. Infected patients with cutaneous symptoms (usually a reflection of vascular alterations) have the highest levels of ACE2 in serum, while on the contrary they have the lowest levels of anti-S IgG1 antibodies. In contrast, patient with respiratory symptoms, especially those with pneumonia have low ACE2 levels but high anti-S antibodies consistent with the overall negative correlation between ACE2 and anti-S. Upper respiratory milder symptoms are intermediate with respect to both ACE2 and anti-S antibodies, while gastrointestinal symptoms are characterized by low ACE2 levels and intermediate antibody levels. Those results indicate that sACE2 and IgG1 anti-S antibody levels may be helpful in discriminating some symptoms. In other words, the levels of ACE2 can be indicative the type of organ in which the infection is developing. It is necessary to increase the number of patients with different cutaneous, intestinal or respiratory symptoms to give the study more statistical power. Considering this, the ACE2 levels can be applied to the clinical management of the disease.

A possible caveat in our interpretation is that infected subjects may have suffered a degradation of ACE2 to lower levels than those before the infection due to the complex dynamics of the angiotensin system and degradation by the virus (3) see also (14) for a review. To minimize this possibility, all serum samples from seropositive people were taken at least 50 days after the disappearance of symptoms, a time likely enough for the RAS system, and especially serum ACE2, to reach homeostatic levels as before the infection, a time which is supported by a recent report (21). Moreover, we have analysed samples of unexposed healthy people. In those persons, the distribution of the ACE2 levels varied across the full spectrum of values, suggesting that the low levels of ACE2 in the infected patients are not by-product of the infection.

A previous report found no difference between serum ACE2 levels between infected and uninfected people (22), whereas we have observed differences in our 2 groups of infected and controls. This apparent discrepancy may be due to the fact that we considered highly exposed but uninfected persons, identifying a group of subjects whose ACE2 distribution is different from the general control population. Moreover, in the above-mentioned manuscript the dynamic range and maximum of the assay to quantify ACE2 serum values appear smaller than the assay used by us. Nonetheless, ACE2 expression varies by geographic ancestry (23) what may affect similar ACE2 analysis in other regions of the world.

The role of ACE2 expression levels on SARS-CoV-2 infection severity is still a debated issue (24–26). Membrane bound ACE2 is the main cellular receptor of SARS-COV-2, and it is expected that an increase in its level may enhance the infection. Nevertheless, ACE2 plays an important physiological role by downregulating the pro-inflammatory peptides Ang II and bradykinin and through this action, it protects the lungs from acute inflammation (27). Therefore, it was proposed by several authors that higher levels of ACE2 may alleviate the severity of SARS-CoV-2 infection (28, 29). Furthermore, although it is generally expected that an increase of membrane bound ACE2 levels (mACE2) may favor the virus propagation, mathematical models show that this not need necessarily be the case (14). This is particularly relevant for soluble ACE2 in the serum (sACE2), which may act as a decoy neutralizing factor of infection sequestering SARS-CoV-2 away from mACE2 (30). Additionally, sACE2 levels can cleave circulating AngII reducing disease morbidity by increase the systemic protective effects of ACE2/Ag-(1–7) as a regulator of inflammation.

Studies examining sACE2 as a risk marker for severe COVID-19 are lacking although it was suggested that such studies should be undertaken (26, 31). Several studies observed that sACE2 tends to increase with age and also that men exhibited higher plasma concentrations of sACE2 relative to children and women (26, 32). Those authors interpreted their results as evidence of a positive correlation between sACE2 levels and COVID-19 severity. Although this was not the objective of our studies we found no association of plasma levels of soluble ACE2 neither with sex nor with age in unexposed seronegative subjects nor in the whole patient population. Another manuscript hypothesized that subjects with higher expression level of ACE2 in nasopharyngeal and oropharyngeal cells of infected patients are more vulnerable to develop more severe complications of COVID-19 (33). These discrepancies may be due to the fact that the correlations presented in the above-mentioned manuscripts are weak and more importantly we include in our study infected patients and highly exposed patients. Contrary to the above, our result with serum ACE2 levels support a negative relationship between sACE2 levels and disease severity, as it is also apparent using rodent data on mACE2 in rat lungs, which yield a very strong negative correlation between estimated ACE2 levels and COVID-19 lethality across age and sex, in agreement with a mechanistic model of Covid-19 lethality (14).

We found that soluble recombinant ACE2 as well as serum from seronegative highly exposed subjects are able to neutralize the virus, whereas sera from negative controls showed a great variability, probably due to the dispersion in serum ACE2 levels, with samples ranging from 3 to 40ng/ml. In fact, most samples with less than 10ng/ml, the lower limit found in seronegative highly exposed subjects, were mostly ineffective. This result supports the hypothesis that low serum ACE2 levels is a negative prognostic factor.

Yeung et al. have shown that sACE is promoting rather than inhibiting the entry of the virus in the cells suggesting a proviral role of sACE2 increasing the virus entry (34). However, in agreement with our other reports it have been shown that sACE2 blocks, rather than enhance, the pseudotyped infection in cell cultures or even SARS-CoV2 infection in cell cultures and animal models (35–39). The reasons of the possible discrepancies are unknown but could be related to that the pro-infection effect of sACE2 could be relevant in cells/tissues where TMPRSS is not expressed or the different sources of rsACE2 used. Previous reports have shown the neutralizing ability of rhACE2 *in vitro* cell cultures with reported IC50 values ranging from 12.6 μg/mL for SARS-CoV-2 (35) to 20-40 ng/mL (38) coincident with our higher sACE2 serum values. *In vitro*, discrepancies can be due to the use of different recombinant protein with different degrees of glycosylation (36). More importantly, hsACE2 was able to strongly inhibit *in vivo* (over 90%) SARS-CoV-2 pseudovirus infection and the serum concentrations extrapolated by them (40) were similar than those described here and presented in our sera. Checking the protective effect of ACE2 would support the utility of soluble ACE2 as a therapy Our results are consistent with the recent results that support use of serum ACE2 as therapy [reviewed in (39)] and clinical trials are underway for rhACE2 (41).

On the quantitative side, we have found weak but significant correlation between sACE2 levels and the ability to neutralize in all tested sera, irrespective of their provenience. This correlation can be improved if we take into account the peptide Ang II, which binds ACE2 and may compete with the binding to the spike protein. The two variables may explain little more than one third of the variance of the neutralization values. One explanation may be due to differences in neutralization between active ACE2 and degradation products (likely neutralization inactive) in patients, and that cannot be discriminated with our assays. In this regard, it has been described recently differences in ACE2 degradation in COVID patients (21). Soluble ACE2 is also present in human bronchoalveolar lavage fluid from healthy subjects (42) and may act as decoy avowing productive infection. Thus, it is possible that this ACE2, likely related to serum ACE2 level, is somewhat protecting highly exposed people avoiding the infection to go further. This would also explain why antiviral antibodies are not generated.

HCW on dedicated COVID-19 had a significantly higher seroprevalence than other frontline health-care workers (43). However, some of them still remained seronegative. Recently, some of us reported that two ACE2 single nucleotide polymorphisms (SNPs), known to be associated with higher ACE2 expression and lower COVID-19 susceptibility (44), are over-represented in our cohort of highly exposed seronegative HCW potentially explaining the higher sACE2 expression that we found in our work and the reduced risk of contracting SARS-CoV-2 infection (45). A previous study have analysed the susceptibility of seronegative HCW to infection (46). In those exposed HCW with negative SARS-CoV-2-specific IgG serum titers, specific IgA antibodies were detectable in their nasal fluids, however in that study HCW either tested negative or positive for SARS-CoV-2 by RT-qPCR, whereas in our study all HCW were repeatedly PCR negative. Together, those results support our hypothesis that higher ACE2 levels may protect from productive infection and contribute to a better the understanding of the high transmissibility of SARS-CoV-2 and the unexplained broad differences in the risk of being infected upon viral exposure.

## Data Availability Statement

The raw data supporting the conclusions of this article will be made available by the authors, without undue reservation.

## Ethics Statement

The study protocol was approved by Empireo Internal Committees as well as by Universidad Autonoma de Madrid (CEI-117- 2352). Written informed consent to participate in this study was provided by the participants’ legal guardian/next of kin.

## Author Contributions

MM, MU, PD, and LH conducted the experiments, acquired and analyzed the data; MAL and LG-B provided human biospecimens. HMvS, BA, LG-B, SS-V, UB, and MF analyzed and interpreted the data. DA and UB performed bioinformatics to analyze FCS data. MF and UB conceived and designed this study. MF wrote the manuscript. All authors provided constructive comments and edits, and approved the final version of this manuscript.

## Funding

This research was funded by grants from “Ministerio de Ciencia e Innovación” (SAF2013-42850-R, SAF2016-75988-R and PID-2019104760RB-100; FEDER), “Comunidad de Madrid (S2017/BMD-3671. INFLAMUNE-CM; FEDER) to MF, Consejo Superior de Investigaciones Científica, CSIC (CSIC-COV19-108, SGL210235) to MF and UB, CRUE-Supera COVID, the European Development Regional Fund ‘‘A way to achieve Europe’’ (ERDF), Merck, Sharp & Dohme Investigator Studies Program (code MISP# IIS 60257), and Fondo Supera COVID-19 (2020-001) to SS-V. Institutional grants from “Fundación Ramón Areces” and “Banco de Santander”. This research work was also funded by the European Commission – NextGenerationEU (Regulation EU 2020/2094), through CSIC's Global Health Platform (PTI Salud Global).

## Conflict of Interest

The authors declare that the research was conducted in the absence of any commercial or financial relationships that could be construed as a potential conflict of interest.

## Publisher’s Note

All claims expressed in this article are solely those of the authors and do not necessarily represent those of their affiliated organizations, or those of the publisher, the editors and the reviewers. Any product that may be evaluated in this article, or claim that may be made by its manufacturer, is not guaranteed or endorsed by the publisher.

## Supplementary Material

Supplementary Figure 1 Serum AngII levels and susceptibility to infection. **(A)** AngII serum levels determined by ELISA in the indicated patient groups.

Supplementary Figure 2 ADAM17 levels and susceptibility to infection. **(A)** Soluble ADAM17 serum levels determined by ELISA in the indicated patient groups.

Supplementary Table 1 Clinical, Demographic Characteristics and individual sACE2, AngII and Anti-S IgG1 antibodies of the subjects in the study.
