# Supplementary figures and images for: ACE2 Serum Levels as Predictor of Infectability and Outcome in COVID-19
Source: Front Immunol. 2022 Mar 23;13:836516. doi: 10.3389/fimmu.2022.836516 (PMC8986157; doi:10.3389/fimmu.2022.836516)

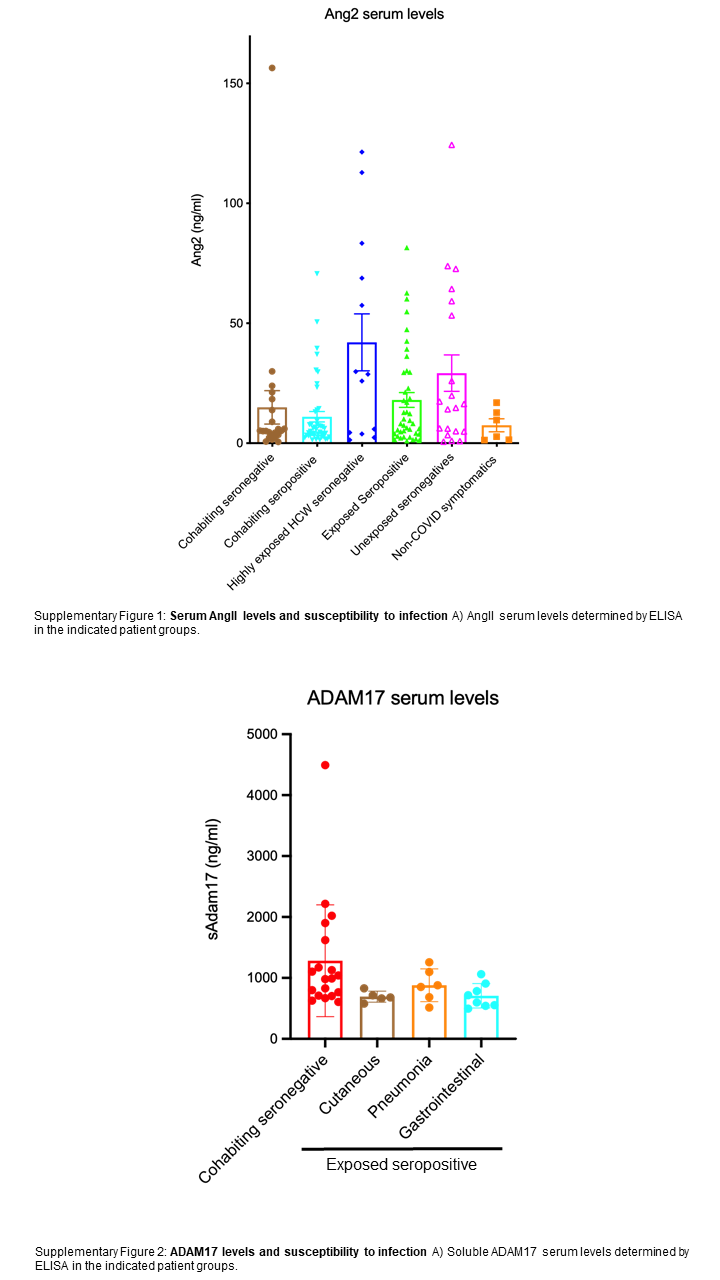

Supplement: Supplementary Figure 1 — Serum AngII levels and susceptibility to infection. (A) AngII serum levels determined by ELISA in the indicated patient groups. [file Image_1.tif]
