# Supplementary material for: ACE2 Serum Levels as Predictor of Infectability and Outcome in COVID-19
Source: Front Immunol. 2022 Mar 23;13:836516. doi: 10.3389/fimmu.2022.836516 (PMC8986157; doi:10.3389/fimmu.2022.836516)
Supplement: Supplementary Table 1 — Clinical, Demographic Characteristics and individual sACE2, AngII and Anti-S IgG1 antibodies of the subjects in the study. [file Table_1.pdf]

Cod	Age	Sex	Classification	Severity of symptoms	Type of symptoms	ACE2 (ng/ml)	anti S IgG1 antibodies (mean of fluorescence)	Ang2 (pg/ml)	ACE2/Ang2 ratio
849	49	F	Exposed Seropositive	Asymptomatics	Asymptomatics	5,68	1082	9,52	0,60
824	41	M	Exposed Seropositive	Asymptomatics	Asymptomatics	16,56	146	3,82	4,33
210	54	F	Exposed Seropositive	Asymptomatics	Asymptomatics	20,46	2990	29,49	0,69
CC42	44	F	Exposed Seropositive	Asymptomatics	Asymptomatics	na	2698	1,54	
174	49	M	Exposed Seropositive	MILD	UR1	6,52	442	22,92	0,28
819	87	F	Exposed Seropositive	MILD	UR1	14,09	2089	1,82	7,73
138	36	F	Exposed Seropositive	MILD	UR1	14,65	1080	4,20	3,49
1068	33	M	Exposed Seropositive	MILD	Gastrointestinal	2,13	345	18,69	0,11
1115	59	F	Exposed Seropositive	MILD	Gastrointestinal	2,48	1046	6,07	0,41
1139	58	M	Exposed Seropositive	MILD	Gastrointestinal	3,52	1887	12,33	0,29
296	57	M	Exposed Seropositive	MILD	UR1	5,5	1169	42,55	0,13
253	27	M	Exposed Seropositive	MILD	UR1	6,48	1098	5,20	1,25
190	47	M	Exposed Seropositive	MILD	UR1	7,55	805	29,61	0,25

198	33	F	Exposed Seropositive	MILD	UR1	8,75	133	8,14	1,07
CBM1	37	F	Exposed Seropositive	MILD	UR1	10,8	49,2	na	
242	48	M	Exposed Seropositive	MILD	UR1	10,89	75,1	62,64	0,17
158	47	M	Exposed Seropositive	MILD	UR1	11,65	320	39,24	0,30
PE12	41	F	Exposed Seropositive	MILD	UR1	12,8	1938	1,01	12,71
244	40	M	Exposed Seropositive	MILD	UR1	13,62	1442	21,31	0,64
NS39	43	F	Exposed Seropositive	MILD	UR1	16,73	1376	1,16	14,42
241	60	M	Exposed Seropositive	MILD	UR1	19,32	2022	47,39	0,41
172	52	M	Exposed Seropositive	MODERATE	Cutaneous	10,16	1333	81,58	0,12
682	29	F	Exposed Seropositive	MODERATE	Cutaneous	10,89	1131	2,91	3,75
1081	41	M	Exposed Seropositive	MODERATE	Gastrointestinal	1,7	589	36,25	0,05
569	53	M	Exposed Seropositive	MODERATE	Gastrointestinal	3,17	1625	7,48	0,42
877	40	F	Exposed Seropositive	MODERATE	Gastrointestinal	5,43	46,8	1,47	3,69
153	48	M	Exposed Seropositive	MODERATE	Gastrointestinal	11,71	1262	30,22	0,39
771	39	M	Exposed Seropositive	MODERATE	Gastrointestinal	14,55	158	8,20	1,77

561	33	F	Exposed Seropositive	MODERATE	Gastrointestinal	18,35	1625	2,55	7,20
785	60	M	Exposed Seropositive	MODERATE	Pneumonia	4,55	2004	4,81	0,95
EM6	54	F	Exposed Seropositive	MODERATE	UR2	10,16	2054	54,82	0,19
161	47	F	Exposed Seropositive	MODERATE	UR2	12,79	738	6,42	1,99
11	54	F	Exposed Seropositive	MODERATE	UR2	17,91	1615	17,06	1,05
176	47	F	Exposed Seropositive	SEVERE	Cutaneous	11,57	464	17,84	0,65
286	42	M	Exposed Seropositive	SEVERE	Cutaneous	15,22	147	60,16	0,25
248	41	F	Exposed Seropositive	SEVERE	Gastrointestinal	4,3	1249	12,84	0,33
690	46	F	Exposed Seropositive	SEVERE	Gastrointestinal	6,9	1292	5,53	1,25
421	54	M	Exposed Seropositive	SEVERE	UR2	4,47	1623	10,12	0,44
834	46	M	Exposed Seropositive	SEVERE	UR2	7,27	2769	5,81	1,25
386	47	F	Exposed Seropositive	SEVERE	Cutaneous	11,01	588	2,18	5,06
MU9	30	F	Exposed Seropositive	SEVERE	Pneumonia	6,82	1670	3,95	1,73
844	57	F	Cohabiting seropositive	Asymptomatics	Asymptomatics	3,64	2398	37,03	0,10
1142	63	F	Cohabiting seropositive	Asymptomatics	Asymptomatics	5,88	1144	3,24	1,81

770	68	F	Cohabiting seropositive	Asymptomatics	Asymptomatics	6,82	1356	1,34	5,08
1127	15	M	Cohabiting seropositive	Asymptomatics	Asymptomatics	9,81	2330	4,33	2,26
1126	12	M	Cohabiting seropositive	Asymptomatics	Asymptomatics	10,05	1540	30,46	0,33
814	37	M	Cohabiting seropositive	Asymptomatics	Asymptomatics	12,34	3601	6,65	1,86
305	14	M	Cohabiting seropositive	Asymptomatics	Asymptomatics	18,87	1239	4,07	4,63
1183	25	F	Cohabiting seropositive	MILD	Cutaneous	14,73	549	3,82	3,86
813	55	M	Cohabiting seropositive	MILD	UR1	3,18	2675	3,07	1,04
812	75	M	Cohabiting seropositive	MILD	UR1	6,35	2508	2,45	2,59
818	63	M	Cohabiting seropositive	MILD	UR1	8,41	1012	14,38	0,58
128	18	F	Cohabiting seropositive	MILD	UR1	17,24	842	3,00	5,75
517	68	F	Cohabiting seropositive	MILD	Gastrointestinal	6,73	2193	3,29	2,04
2	65	F	Cohabiting seropositive	MILD	Gastrointestinal	7,91	1685	4,60	1,72
225	37	M	Cohabiting seropositive	MILD	UR1	5,77	2188	3,74	1,54
786	50	F	Cohabiting seropositive	MILD	UR1	7,31	971	7,83	0,93
1049	54	F	Cohabiting seropositive	MILD	UR1	9,57	2715	7,03	1,36

984	68	F	Cohabiting seropositive	MILD	UR1	9,69	1787	1,75	5,54
245	34	M	Cohabiting seropositive	MILD	UR1	10,17	2977	5,70	1,78
MU10	27	M	Cohabiting seropositive	MILD	UR2	5,5	573	4,93	1,12
603	44	M	Cohabiting seropositive	MODERATE	Gastrointestinal	4,04	552	7,23	0,56
127	50	F	Cohabiting seropositive	MODERATE	Gastrointestinal	4,79	559	2,46	1,95
821	62	M	Cohabiting seropositive	MODERATE	Gastrointestinal	7,31	1262	4,10	1,78
1034	61	M	Cohabiting seropositive	MODERATE	Gastrointestinal	11,15	1472	9,25	1,21
278	38	F	Cohabiting seropositive	MODERATE	Gastrointestinal	16,32	872	2,29	7,14
252	39	M	Cohabiting seropositive	MODERATE	Gastrointestinal	18,25	1149	7,71	2,37
782	43	F	Cohabiting seropositive	MODERATE	UR2	4,32	2515	50,56	0,09
799	35	M	Cohabiting seropositive	MODERATE	UR2	6,99	1584	4,13	1,69
1734	35	M	Cohabiting seropositive	SEVERE	Gastrointestinal	2,13	1802	23,28	0,09
622	67	F	Cohabiting seropositive	SEVERE	Gastrointestinal	2,57	2041	2,64	0,97
624	59	F	Cohabiting seropositive	SEVERE	Gastrointestinal	2,65	1184	7,83	0,34
277	40	M	Cohabiting seropositive	SEVERE	Gastrointestinal	3,78	1417	13,08	0,29

735	28	F	Cohabiting seropositive	SEVERE	Gastrointestinal	4,04	658	3,81	1,06
852	50	M	Cohabiting seropositive	SEVERE	Gastrointestinal	8,18	3114	6,17	1,33
1050	45	F	Cohabiting seropositive	SEVERE	Gastrointestinal	10,05	185	6,74	1,49
518	70	M	Cohabiting seropositive	SEVERE	Gastrointestinal	13,75	2282	13,79	1,00
820	64	F	Cohabiting seropositive	SEVERE	Pneumonia	7,19	4145	2,55	2,82
742	49	M	Cohabiting seropositive	SEVERE	Pneumonia	7,43	1942	70,63	0,11
275	66	F	Cohabiting seropositive	SEVERE	Gastrointestinal	4,47	2066	1,56	2,87
1128	55	M	Cohabiting seropositive	SEVERE	Pneumonia	8,14	3418	6,09	1,34
8	na	na	Cohabiting seropositive	na	na	6,238	2644	29,69	0,21
9	na	na	Cohabiting seropositive	na	na	6,595	1755	24,72	0,27
1	na	na	Cohabiting seropositive	na	na	9,33	2769	39,51	0,24
740	na	na	Cohabiting seropositive	na	na	9,333	2667	1,63	5,74
739	na	na	Cohabiting seropositive	na	na	13,38	2769	1,40	9,54
169	54	F	Highly exposed HCW seronegative	MODERATE	Cutaneous	15,86	345	13,03	1,22

151	47	M	Highly exposed HCW seronegative	MILD	Cutaneous	15,48	130	83,36	0,19
126	63	F	Highly exposed HCW seronegative	MILD	Cutaneous	24,95	225	1,33	18,83
199	45	F	Highly exposed HCW seronegative	Asymptomatics	Asymptomatics	16,81	149	3,85	4,37
206	41	F	Highly exposed HCW seronegative	Asymptomatics	Asymptomatics	15,32	93,8	5,85	2,62
109	40	M	Highly exposed HCW seronegative	Asymptomatics	Asymptomatics	24,35	122	4,42	5,51
PM	43	M	Highly exposed HCW seronegative	Asymptomatics	Asymptomatics	27,32	104	2,38	11,47
RYC14	31	F	Highly exposed HCW seronegative	Asymptomatics	Asymptomatics	10,048	143	na	
RYC15	53	F	Highly exposed HCW seronegative	Asymptomatics	Asymptomatics	10,048	190	na	
RYC18	50	F	Highly exposed HCW seronegative	Asymptomatics	Asymptomatics	13,857	190	na	
RYC21	57	F	Highly exposed HCW seronegative	Asymptomatics	Asymptomatics	11	150	na	

RYC24	34	F	Highly exposed HCW seronegative	Asymptomatics	Asymptomatics	11,476	255	na	
RYC25	36	M	Highly exposed HCW seronegative	Asymptomatics	Asymptomatics	16,476	164	121,38	0,14
RYC43	48	F	Highly exposed HCW seronegative	Asymptomatics	Asymptomatics	12,19	177	na	
RYC44	48	F	Highly exposed HCW seronegative	Asymptomatics	Asymptomatics	10,405	148	68,82	0,15
RYC45	51	F	Highly exposed HCW seronegative	Asymptomatics	Asymptomatics	11,595	158	na	
RYC46	42	F	Highly exposed HCW seronegative	Asymptomatics	Asymptomatics	11,476	205	na	
RYC47	35	M	Highly exposed HCW seronegative	Asymptomatics	Asymptomatics	13,857	164	na	
RYC50	30	M	Highly exposed HCW seronegative	Asymptomatics	Asymptomatics	12,548	159	na	
RYC51	38	F	Highly exposed HCW seronegative	Asymptomatics	Asymptomatics	11,476	160	na	
RYC52	28	F	Highly exposed HCW seronegative	Asymptomatics	Asymptomatics	23,976	1151	na	

RYC53	40	F	Highly exposed HCW seronegative	Asymptomatics	Asymptomatics	14,333	188	29,83	0,48
RYC54	44	F	Highly exposed HCW seronegative	Asymptomatics	Asymptomatics	16,238	191	na	
RYC55	55	M	Highly exposed HCW seronegative	Asymptomatics	Asymptomatics	11,357	332	na	
RYC56	28	F	Highly exposed HCW seronegative	Asymptomatics	Asymptomatics	10,405	202	na	
RYC57	37	F	Highly exposed HCW seronegative	Asymptomatics	Asymptomatics	14,571	186	25,93	0,56
RYC58	40	M	Highly exposed HCW seronegative	Asymptomatics	Asymptomatics	13,857	538	na	
RYC60	37	F	Highly exposed HCW seronegative	Asymptomatics	Asymptomatics	14,214	183	na	
RYC62	36	F	Highly exposed HCW seronegative	Asymptomatics	Asymptomatics	12,31	279	na	
RYC63	49	F	Highly exposed HCW seronegative	Asymptomatics	Asymptomatics	16,238	225	na	
RYC64	49	F	Highly exposed HCW seronegative	Asymptomatics	Asymptomatics	12,429	176	28,76	0,43

RYC65	44	F	Highly exposed HCW seronegative	Asymptomatics	Asymptomatics	14,095	961	na	
RYC66	57	F	Highly exposed HCW seronegative	Asymptomatics	Asymptomatics	18,857	151	na	
RYC69	41	M	Highly exposed HCW seronegative	Asymptomatics	Asymptomatics	13,381	164	112,85	0,12
RYC70	35	F	Highly exposed HCW seronegative	Asymptomatics	Asymptomatics	9,929	133	na	
RYC71	38	F	Highly exposed HCW seronegative	Asymptomatics	Asymptomatics	15,643	140	57,46	0,27
RYC72	58	F	Highly exposed HCW seronegative	Asymptomatics	Asymptomatics	13,381	167	na	
784	48	F	Cohabiting seronegative	MODERATE	UR2	12,5	154	2,65	4,71
1077	66	M	Cohabiting seronegative	--	--	9,93	1947	156,43	0,06
769	34	F	Cohabiting seronegative	--	--	10,23	191	13,80	0,74
970	10	M	Cohabiting seronegative	--	--	10,64	235	0,53	20,06
713	41	M	Cohabiting seronegative	--	--	10,88	235	23,89	0,46
801	48	M	Cohabiting seronegative	--	--	11,14	180	8,88	1,25

822	29	M	Cohabiting seronegative	--	--	11,36	189	3,50	3,25
787	52	M	Cohabiting seronegative	--	--	11,83	127	21,24	0,56
853	49	F	Cohabiting seronegative	--	--	12,43	120	5,15	2,41
EF1	60	F	Cohabiting seronegative	--	--	14,08	142	29,91	0,47
738	45	F	Cohabiting seronegative	--	--	14,33	587	1,51	9,47
779	64	M	Unexposed seronegative	--	--	15	136	18,39	0,82
211	35	M	Cohabiting seronegative	--	--	15,68	188	5,23	3,00
748	69	M	Cohabiting seronegative	--	--	15,83	64,8	5,05	3,14
322	44	F	Cohabiting seronegative	--	--	16,57	185	3,82	4,34
823	33	F	Cohabiting seronegative	--	--	18,45	1296	0,70	26,45
816	54	M	Cohabiting seronegative	--	--	19,47	153	6,01	3,24
179	56	F	Cohabiting seronegative	--	--	20,73	74,7	4,38	4,73
129	14	M	Cohabiting seronegative	--	--	21,59	167	5,90	3,66
110	34	F	Cohabiting seronegative	--	--	22,46	136	2,27	9,88
304	17	M	Cohabiting seronegative	--	--	24,46	265	5,01	4,89

845	66	M	Cohabiting seronegative	--	--	24,82	966	2,03	12,20
303	50	F	Cohabiting seronegative	--	--	33	129	4,56	7,23
746	38	M	Non-COVID sympytomatics	--	--	5,17	50,4	16,87	0,31
743	23	F	Non-COVID sympytomatics	--	--	10,17	200	1,41	7,23
679	35	F	Non-COVID sympytomatics	--	--	10,29	185	12,72	0,81
817	20	F	Non-COVID sympytomatics	--	--	13,41	174	2,75	4,87
NS40	46	M	Non-COVID sympytomatics	--	--	14,35	94,3	9,65	1,49
687	71	M	Non-COVID sympytomatics	--	--	15,45	181	1,30	11,91
854	33	F	Unexposed seronegative	--	--	3,18	195	0,64	5,00
768	49	F	Unexposed seronegative	--	--	4,13	45,7	0,88	4,71
847	62	M	Unexposed seronegative	--	--	5,45	268	59,24	0,09
851	34	M	Unexposed seronegative	--	--	6,82	162	14,12	0,48
850	26	M	Unexposed seronegative	--	--	7,27	199	124,37	0,06
628	39	M	Unexposed seronegative	--	--	7,79	215	6,17	1,26
CBM2	68	F	Unexposed seronegative	--	--	8,29		na	

765	33	F	Unexposed seronegative	--	--	8,46	54,7	4,83	1,75
781	78	F	Unexposed seronegative	--	--	9,55	10,2	3,37	2,83
968	46	M	Unexposed seronegative	--	--	9,81	175	73,84	0,13
CBM3	30	F	Unexposed seronegative	--	--	10,2		na	
848	62	F	Unexposed seronegative	--	--	11,14	165	0,99	11,29
95	64	M	Unexposed seronegative	--	--	13,11	107	64,34	0,20
CBM4	30	F	Unexposed seronegative	--	--	13,49		na	
969	14	M	Unexposed seronegative	--	--	15,36	168	6,08	2,53
230	56	M	Unexposed seronegative	--	--	15,81	180	53,23	0,30
676	52	F	Unexposed seronegative	--	--	15,91	162	16,32	0,97
778	66	F	Unexposed seronegative	--	--	15,91	140	4,99	3,19
243	40	M	Unexposed seronegative	--	--	17,43	76,1	72,71	0,24
42	na	M	Unexposed seronegative	--	--	19,65	93,3	25,94	0,76
388	13	M	Unexposed seronegative	--	--	32,38	83,2	19,82	1,63
132	56	F	Unexposed seronegative	--	--	34,16	117	17,35	1,97

747	29	M	Unexposed seronegative	--	--	38,9	99	14,75	2,64
